# The Relationship between Sleep-Disordered Breathing and Hypertension in a Nationally Representative Sample

**DOI:** 10.1155/2015/769798

**Published:** 2015-04-29

**Authors:** Sarah Dee Geiger, Anoop Shankar

**Affiliations:** ^1^Northern Illinois University, School of Nursing and Health Studies, DeKalb, IL 60115, USA; ^2^8816 Manchester Road No. 159, St. Louis, MO 63144, USA

## Abstract

Sleep-disordered breathing (SDB), characterized by abnormal respiratory patterns or inadequate quantity of ventilation, is common in adults. A positive association between SDB and hypertension has been established, in both cross-sectional and longitudinal studies. One void in the literature concerns the role of race/ethnicity in the association between SDB and hypertension. In this context, a cross-sectional study was performed on 6,783 participants in the National Health and Nutrition Examination Survey 2005–2008. Participants were ≥age 20 and free from cardiovascular disease. The outcome of interest was hypertension, defined as ≥140 mmHg systolic blood pressure (BP), and/or ≥90 mmHg diastolic BP or antihypertensive medication use. Self-reported SDB was positively associated with hypertension, independent of confounders such as depression, diabetes, cholesterol levels, and body mass index, among others. The association persisted in subgroup analyses by gender, with a stronger association among males than females, as well as by race/ethnicity, with non-Hispanic blacks displaying the strongest association. In the multivariable-adjusted model, compared to a sleep summary score of zero (referent), the OR (95% CI) of hypertension for non-Hispanic blacks was 1.34 (0.98–1.83) for a sleep summary score of 1, 1.44 (1.06–1.97) for a score of 2 and 3.72 (1.98–7.00) for a score of >3; *p-trend* < 0.0001. SDB was positively associated with hypertension in a large, nationally representative sample of US adults. Along with being prevalent, SDB is also treatable. Therefore, our results are important for minority race/ethnic groups who typically experience a higher baseline for negative health outcomes.

## 1. Introduction

Sleep-disordered breathing (SDB) is common in adults in developed nations throughout the United States, Europe and Australia [1–3]. SDB is an umbrella term for a number of sleep disorders characterized by abnormal respiratory patterns or inadequate quantity of ventilation. Some indicators or markers of SDB are sleep apnea, hypopnea (characterized by overly shallow breathing), snoring, snorting, daytime sleepiness, undersleeping, and oversleeping [4–8]. There are many reasons for the growing prevalence of sleep disorder indicators, including the invention of artificial light, the rise of shift work, and 24-hour manufacturing.

Some risk factors for SDB have been identified, including age, gender, and body mass index (BMI) [9, 10]. Similarly, the association between SDB and hypertension has been established, in both cross-sectional and longitudinal studies [7, 11–14]. Health consequences of hypertension range from manageable to grave, and the condition may potentiate other, more severe health outcomes such as compromised cardiac, arterial, and/or renal function [15]. In those instances where SDB is treatable, more dire health consequences such as hypertension may be largely avoidable [11].

One void in the literature concerns the role of race/ethnicity in the association between SDB and hypertension. To address this question, we used a large, nationally representative, multiethnic database to examine the relationship between SDB and hypertension, both overall and in several subanalyses, including race/ethnicity. The study aims to provide further evidence for the overall association, while uncovering trends in specific understudied demographics.

## 2. Methods

### 2.1. Study Population

This study uses merged data from the National Health and Nutrition Examination Survey (NHANES), 2005–2008, which is a multiethnic, nationally representative sample, excluding institutionalized civilians. The data collection methods for NHANES have been published and are available online [16]. The study sample is a stratified multistage probability sample selected from counties, census blocks, households, and individuals therein. Non-Hispanic blacks and Mexican Americans were oversampled to provide true representations of those races/ethnicities. NHANES respondents provided written consent for participation and the US Department of Health and Human Service's Human Subjects Committee approved the study. Self-reported sleep variables include sleep duration, snoring, snorting/stop breathing, sleep apnea, and daytime sleepiness; for the current study, we restricted the sample to adults >20 years of age who took part in both the interview and examination components (*n* = 10,914 remaining). We also excluded those with missing values for all covariates, including all sleep variables, education level, BMI, serum and fasting glucose, and depression (*n* = 7,562 remaining). Because of its established potential for association with the indicator and outcome variables, those with CVD were also excluded from analysis, for a final sample size of (*n* = 6,783 remaining).

### 2.2. Main Outcome of Interest: Hypertension

Participants could qualify as hypertensive in three ways: physician diagnosis, taking hypertension medication, or high blood pressure. The first two possibilities were self-reported in the interview portion of the survey, whereas blood pressure was measured in the examination. Blood pressure examination was denied if presence of the following was observed on both arms: rashes, gauze dressings, casts, edema, paralysis, tubes, open sores or wounds, withered arms, a-v shunts, blood had been drawn from arm within last week, or arms too large for the blood pressure cuff. After determining pulse, three seated systolic/diastolic blood pressure measurements were taken using a mercury sphygmomanometer by the examining physician following a strict protocol as recommended by the American Heart Association. For the current paper, we used an average of the three measurements to determine high blood pressure, with cutoffs of >140 for mean systolic and > 90 for mean diastolic.

### 2.3. Exposure Measurements

Beginning in 2005, NHANES introduced a questionnaire that evaluates sleep habits using items validated in existing research [17]. SDB was assessed through questionnaire items such as, “how much sleep do you usually get at night on weekdays or workdays?,” “in the past 12 months, how often did you snore while you were sleeping?,” “in the past 12 months, how often did you snort, gasp, or stop breathing while you were asleep?,” and “in the past month, how often did you feel excessively or overly sleepy during the day [18]?” Using participant responses to these questions we created a summary sleep score or sleep index, including the variables “sleep duration,” “snoring,” “snorting,” and “daytime sleepiness.” The daytime sleepiness variable was categorized into “never or rare,” “sometimes,” and “often or almost always,” with sometimes meaning 2–4 times per month and often or almost always being 5+ times per month. Snorting and snoring were put into “never or rare,” “occasional,” and “frequent” categories, where occasional is defined as 3-4 nights per week and frequent as 5+ nights per week. We categorized the “sleep duration” variable into a gradient of <5, 6, 7, 8, or >9 hours, as dictated by the survey questionnaire [6, 18, 19].

To calculate the SDB summary score, we dichotomized the created variables (sleep duration, snoring, snorting, and daytime sleepiness) and assigned each variable a point, respectively, if: sleep duration >5 hours, snoring and/or snorting a minimum of 3-4 times/week, and experiencing daytime sleepiness a minimum of 5 times/month. We also evaluated sleep apnea separate from the SDB summary score, based on the NHANES survey questions, “have you ever been told by a doctor or other health professional that you have a sleep disorder?” (yes or no), followed by “what was the sleep disorder? Check all that apply.” (sleep apnea, insomnia, restless legs, and others) [18].

Covariates gender, age, race/ethnicity, education level, smoking status, alcohol intake, physical activity, BMI, history of depression, and history of diabetes were assessed by interview. Total cholesterol and C-reactive protein measurements were taken by blood draw. BMI was calculated by weight (kg)/height (m^2^).

Respondents were considered smokers if they answered affirmatively to the question, “do you smoke now?” Those who answered negatively but reported smoking a total of >100 cigarettes ever were categorized as former smokers, whereas respondents who reported smoking <100 total cigarettes throughout their lifetime were considered never smokers. Moderate physical activity was defined as participation in any moderate recreational physical activity. Individuals were classified as depressed if they self-reported feeling down/depressed/hopeless for more than half-days, nearly every day throughout the prior two weeks.

Details of blood collection and analysis are provided in the NHANES Laboratory/Medical Technologists Procedures Manual [20]. Briefly, serum total cholesterol was measured enzymatically and high-sensitivity CRP measurements were obtained using latex-enhanced nephelometry. The American Diabetes Association definition was used to identify diabetes cases: fasting serum glucose level of >126 mg/dL, nonfasting serum glucose level of >200 mg/dL, or self-reported current oral hypoglycemic medication or insulin use.

### 2.4. Statistical Analysis

In this study, the primary indicator variable was summary sleep score, although the sleep score components were considered individually as well. After checking the distributions of all indicator and outcome variables and covariates, we performed additional descriptive statistics. Multivariable-adjusted logistic regression models were then conducted to examine the association between SDB-related factors and hypertension. Using hypertension as the outcome variable, logistic regressions were then conducted using sleep duration, snoring, snorting, daytime sleepiness, and summary sleep score as independent variables; odds ratios (OR, 95% CI) were calculated for each response category, with the lowest OR serving as the referent category for each variable. We used two regression models, each building on the previous one. The first model is age (years) and sex (male, female) adjusted, and the second model adds demographic, lifestyle, and other risk factors race/ethnicity (non-Hispanic whites, non-Hispanic blacks, Mexican Americans, and others), education (below high school, high school, and above high school), smoking (never smoker, former smoker, and current smoker) and alcohol intake (absent, present), physical activity (moderate physical activity), body mass index (obese, nonobese), depression (absent, present), diabetes (absent, present), total cholesterol (mg/dL), and C-reactive protein (mg/dL), which have the potential to act as intermediaries between SDB indicators and hypertension. To evaluate consistency of the association between SDB-related factors and hypertension, we conducted additional analyses, stratified by sex (men, women) and race/ethnicity (non-Hispanic whites, non-Hispanic blacks, and Mexican Americans and others). Using SAS (version 9.2; SAS Institute, Cary, NC) and SUDAAN (version 8.0; Research Triangle Institute, Research Triangle Park, NC) software, sample weights were employed in all models to account for unequal selection, oversampling, and nonresponse of some groups.

## 3. Results

Table 1 presents the characteristics of the study population. Among 6,783 Appalachian adults > 20 years of age, 2,351 were hypertensive. About half of those with hypertension were women and the average age for hypertensive individuals was 53.6 years. About half of the hypertensive participants were former or current smokers compared to about 45% of nonhypertensive, an insignificant difference (*p* = 0.52). About 68% of those with hypertension reported being current drinkers as opposed to about 77% in the nondisease group. Hypertensive individuals in this sample tend to exhibit higher BMI than those without the condition, with 78.5% being overweight or obese compared to 61.5% of those with no hypertension. About 18% of those with hypertension also received a diabetes classification, versus 4.6% of those without hypertension, a highly significant difference (*p* < 0.0001). In Table 1, all differences between hypertension and no hypertension were significant except for smoking status.

Table 2 provides associations between SDB markers and presence of hypertension for the overall cohort. Increasing categories of snoring (nights/week in the past year), snorting (nights/week in the past year), daytime sleepiness (times/month), and SDB summary score were positively associated with hypertension in the age and sex-adjusted model. However, after multivariable adjustment for confounders, only short sleep duration, snoring, and the summary SDB score were associated with hypertension.

Table 3 presents the association between increasing SDB summary score and hypertension, stratified by gender. Here also in the model, there was a strong, positive association between increasing SDB summary score and hypertension. However, with multivariable adjustment for confounders, the association persisted only in men; the association in women was substantially attenuated and lost statistical significance.

Table 4 presents the association between SDB summary score and hypertension, stratified by three race/ethnicity categories (non-Hispanic whites, non-Hispanic blacks, and Mexican Americans and others). Here, we found that increasing SDB summary score was positively associated with hypertension in all race/ethnicity categories in both the age, sex-adjusted model and the multivariable-adjusted model. However, the initial strong magnitude of association in the age, sex-adjusted model was substantially attenuated after multivariable adjustment in non-Hispanic whites and Mexican Americans and others; in contrast, the magnitude of association continued to be strong in non-Hispanic blacks even after multivariable adjustment.

## 4. Discussion

We found an overall significant positive association between SDB markers and hypertension in a nationally representative sample of adults. The relationship was found to be independent of prominent hypertension risk factors. In subgroup analyses by gender and race/ethnicity, we found that the SDB-hypertension association was present only in men and not women but was present in all race/ethnicity groups. These results are in keeping with the current findings about the relationship between SDB and hypertension and introduce a new finding concerning its persistence across race/ethnicity [11, 21–23].

### 4.1. Internal Validity

Our principal finding, the positive association between SDB and hypertension, possesses high internal validity, demonstrated by several factors. First, the association is independent from traditional risk factors such as sex, age, race-ethnicity, education level, employment status, and income. Second, results indicate a dose-response relationship between SDB and hypertension. Third, the association persists across stratified race/ethnicity subgroups. The final indicator of high internal validity is strength of the association, which persisted across all models. These points support the conclusion that our findings are not due to chance.

### 4.2. Contribution to Extant Literature

The results of this study are consistent with published studies reporting a positive association between SDB and hypertension [11, 21–24]. One novel finding is the persistent association among only men in the multivariable-adjusted model. Also, our results are unique in the fact that they stem from a comprehensive national survey using merged data from several years, allowing us to accurately examine the putative association among racial/ethnic groups. The size and power of our sample allowed us to assess the relationship across several models and also to conduct separate analyses by gender and race/ethnicity.

Men in the US are at elevated risk for cardiovascular disease overall, including hypertension, compared with American women; in 2009-10, men experienced an age-adjusted hypertension prevalence of 29.4% compared to 27.5% for women [25]. In our study, women and men both experienced a significant association between SDB and hypertension in the age, sex-adjusted model, but after controlling for additional lifestyle risk factors, the association persisted only in men. The current study therefore suggests that the association in women may be due to lifestyle and behavioral factors such as BMI, smoking, alcohol consumption, and lack of physical activity, whereas men may experience a residual effect of SDB independent of those factors.

Ours is the first study examining the relationship between SDB and hypertension to present subgroup analyses by race/ethnicity. Although the association was significant across all race/ethnic groups, the association was diminished upon multivariable adjustment among the nonblack groups. Strong significance persisted among blacks even after multivariable adjustment, indicating that SDB has a residual effect in blacks independent of lifestyle risk factors as well as other hypertension risk factors such as total cholesterol C-reactive protein levels, and diabetes.

### 4.3. Strengths/Limitations

This study's primary strength is use of a multiethnic sample that is representative of the US population. Other advantages are standardized data collection methods and the sizeable resources allocated by NHANES to collect, clean, and check for data accuracy. Comprehensiveness of the NHANES data also allowed us to control for possible confounders and risk factors in regression models.

Study limitations should also be noted when interpreting the results. Foremost, the study is cross-sectional, so the results cannot be interpreted as causal. Second, the use of subjective SDB markers may introduce measurement error and therefore misclassification bias as opposed to using the gold standard polysomnography to evaluate SDB. Nonetheless, this misclassification bias is likely to be nondifferential and likely to bias our OR estimates toward the null and would only cause our interpretations to be conservative.

## 5. Conclusion

Our results showed a positive association between SDB markers and presence of hypertension in a nationally representative, multiethnic sample of US adults. The unique nature of our study is the report that the SDB-hypertension association was present only in men in the gender subgroup analysis and that the association was consistently present in all race/ethnic groups. Along with being prevalent in US adults, SDB is also treatable. Therefore, our results are important for minority race/ethnic groups who typically experience a higher baseline for negative health outcomes.

## Figures and Tables

**Table 1:** Baseline characteristics of the study population^*^.

Characteristics	No hypertension (*N* = 4432)	Hypertension (*N* = 2351)	*p* value
Women (%)	2313 (51.9)	1145 (50.1)	0.011
Age (years)	40.2 ± 0.4	53.6 ± 0.6	<0.0001
Race/ethnicity (%)			<0.0001
Non-Hispanic whites	2123 (71.1)	1210 (74.8)	
Non-Hispanic blacks	765 (9.0)	587 (12.4)	
Mexican Americans	973 (9.4)	327 (5.2)	
Others	571 (10.4)	227 (7.4)	
Education categories (%)			<0.0001
Below high school	1121 (16.0)	687 (18.7)	
High school	1049 (23.7)	590 (25.7)	
Above high school	2262 (60.2)	1074 (55.6)	
Smoking (%)			0.522
Never smoker	2479 (54.6)	1188 (50.1)	
Former smoker	905 (21.0)	697 (29.2)	
Current smoker	1048 (24.3)	466 (20.7)	
Alcohol intake (%)			<0.0001
Current drinker	3164 (76.7)	1441 (68.4)	
Moderate physical activity (%)	2168 (55.6)	1005 (49.6)	<0.0001
Body mass index (%)			<0.0001
Normal weight	1568 (38.5)	483 (21.4)	
Overweight	1595 (34.3)	781 (32.9)	
Obese	1269 (27.2)	1087 (45.6)	
Depression (%)	262 (4.5)	172 (6.4)	0.023
Diabetes (%)	287 (4.6)	544 (18.0)	<0.0001
Total cholesterol (mg/dL)	198.75 ± 0.79	204.34 ± 1.25	0.002
C-reactive protein (mg/dL)	0.364 ± 0.015	0.452 ± 0.019	<0.0001

^*^Data presented are number (percentages) or mean values ± standard error (SE), as appropriate for the variable.

**Table 2:** Association between sleep variables and hypertension.

Sleep variables	Sample size (hypertension %)	Age, sex adjusted odds ratio (95% CI)	Multivariable-model^†^ odds ratio (95% CI)
Sleep duration (hours)			
≤5 hrs	1025 (36.0)	1.62 (1.34–1.96)	1.31 (1.08–1.59)
6 hrs	1551 (32.2)	1.20 (1.01–1.43)	1.15 (0.97–1.37)
7 hrs	1937 (29.1)	1 (referent)	1 (referent)
8 hrs	1823 (30.9)	1.05 (0.92–1.21)	1.05 (0.91–1.22)
≥9 hrs	447 (32.6)	1.24 (0.88–1.74)	1.25 (0.89–1.76)
*p*-trend		0.0006	0.15
Snoring (nights/week)			
0–2	3367 (25.5)	1 (referent)	1 (referent)
3-4	1283 (33.0)	1.34 (1.13–1.60)	1.17 (0.97–1.41)
≥5	2133 (39.6)	1.72 (1.46–2.02)	1.37 (1.15–1.63)
*p*-trend		<0.0001	0.0005
Snorting (nights/week)			
0–2	5978 (30.1)	1 (referent)	1 (referent)
3-4	420 (38.0)	1.21 (0.93–1.57)	1.05 (0.79–1.39)
≥5	385 (43.0)	1.61 (1.28–2.02)	1.19 (0.92–1.53)
*p*-trend		<0.0001	0.18
Daytime sleepiness (times/month)			
0-1	3873 (31.7)	1 (referent)	1 (referent)
2–4	1763 (29.9)	1.05 (0.89–1.24)	1.01 (0.84–1.20)
≥5	1147 (32.6)	1.36 (1.16–1.59)	1.17 (0.98–1.39)
*p*-trend		0.002	0.18
Summary SDB score			
0	3186 (27.2)	1 (referent)	1 (referent)
1	2388 (33.2)	1.33 (1.12–1.58)	1.18 (0.99–1.41)
2	917 (37.9)	1.71 (1.45–2.02)	1.33 (1.09–1.63)
≥3	292 (44.0)	2.44 (1.87–3.19)	1.72 (1.26–2.33)
*p*-trend		<0.0001	<0.0001

^†^Multivariable model: adjusted for age (years), gender (male, female), race/ethnicity (non-Hispanic whites, non-Hispanic blacks, Mexican Americans, and others), education (below high school, high school, and above high school), smoking (never smoker, former smoker, and current smoker), alcohol intake (absent, present), physical activity (moderate physical activity), body mass index (obese, nonobese), depression (absent, present), diabetes (absent, present), total cholesterol (mg/dL), and C-reactive protein (mg/dL).

**Table 3:** Association between sleep variables and hypertension, by gender.

Sleep summary score	Sample size (hypertension %)	Age-adjusted odds ratio (95% CI)	Multivariable-model 2^†^ odds ratio (95% CI)
Men			
0	1441 (27.6)	1 (referent)	1 (referent)
1	1236 (33.7)	1.28 (1.02–1.60)	1.20 (0.96–1.51)
2	493 (38.0)	1.68 (1.35–2.10)	1.40 (1.07–1.85)
≥3	155 (45.5)	2.49 (1.51–4.08)	2.10 (1.24–3.57)
*p*-trend		<0.0001	0.0001
Women			
0	1745 (26.8)	1 (referent)	1 (referent)
1	1152 (32.7)	1.41 (1.10–1.81)	1.16 (0.90–1.48)
2	424 (37.8)	1.74 (1.26–2.39)	1.22 (0.86–1.75)
≥3	137 (42.1)	2.36 (1.32–4.24)	1.33 (0.68–2.59)
*p*-trend		<0.0001	0.18

^†^Multivariable model: adjusted for age (years), race/ethnicity (non-Hispanic whites, non-Hispanic blacks, Mexican Americans, and others), education (below high school, high school, and above high school), smoking (never smoker, former smoker, and current smoker), alcohol intake (absent, present), physical activity (moderate physical activity), body mass index (obese, nonobese), depression (absent, present), diabetes (absent, present), total cholesterol (mg/dL), and C-reactive protein (mg/dL).

**Table 4:** Association between sleep variables and hypertension, by race/ethnicity.

Sleep summary score	Sample size (hypertension %)	Age, sex adjusted odds ratio (95% CI)	Multivariable-model l 2^†^ odds ratio (95% CI)
Non-Hispanic whites			
0	1590 (28.8)	1 (referent)	1 (referent)
1	1148 (34.6)	1.32 (1.06–1.64)	1.20 (0.96–1.50)
2	448 (38.2)	1.58 (1.25–2.00)	1.24 (0.95–1.62)
≥3	147 (40.2)	2.13 (1.43–3.18)	1.47 (0.92–1.34)
*p*-trend		<0.0001	0.01
Non-Hispanic blacks			
0	576 (32.6)	1 (referent)	1 (referent)
1	502 (40.9)	1.53 (1.16–2.01)	1.34 (0.98–1.83)
2	210 (41.4)	1.67 (1.26–2.20)	1.44 (1.06–1.97)
≥3	64 (65.1)	4.42 (2.31–8.46)	3.72 (1.98–7.00)
*p*-trend		<0.0001	<0.0001
Mexican Americans and others			
0	1020 (17.9)	1 (referent)	1 (referent)
1	738 (22.8)	1.14 (0.79–1.64)	1.00 (0.72–1.38)
2	259 (34.1)	2.30 (1.38–3.84)	1.80 (1.07–3.04)
≥3	81 (45.5)	2.43 (1.40–4.22)	1.71 (1.01–2.90)
*p*-trend		0.0002	0.01

^†^Multivariable model: adjusted for age (years), gender (male, female), education (below high school, high school, and above high school), smoking (never smoker, former smoker, and current smoker), alcohol intake (absent, present), physical activity (moderate physical activity), body mass index (obese, nonobese), depression (absent, present), diabetes (absent, present), total cholesterol (mg/dL), and C-reactive protein (mg/dL).
